# Genome analysis of *Shewanella putrefaciens* 4H revealing the potential mechanisms for the chromium remediation

**DOI:** 10.1186/s12864-024-10031-9

**Published:** 2024-02-02

**Authors:** Yajun Cai, Xu Chen, Hanghang Qi, Fantong Bu, Muhammad Shaaban, Qi-An Peng

**Affiliations:** 1 College of Environmental Engineering, Wuhan Textile University, Wuhan, 430200 China; 2 Clean Production of Textile Printing and Dyeing Engineering Research Center of Ministry of Education, Wuhan, 430200 China; 3 College of Agriculture, Henan University of Science and Technology, Luoyang, China

**Keywords:** Shewanella putrefaciens, Cr(VI) reduction, Genome sequence, Metabolism-related genes

## Abstract

Microbial remediation of heavy metal polluted environment is ecofriendly and cost effective. Therefore, in the present study, *Shewanella putrefaciens* stain 4H was previously isolated by our group from the activated sludge of secondary sedimentation tank in a dyeing wastewater treatment plant. The bacterium was able to reduce chromate effectively. The strains showed significant ability to reduce Cr(VI) in the pH range of 8.0 to 10.0 (optimum pH 9.0) and 25–42 ℃ (optimum 30 ℃) and were able to reduce 300 mg/L of Cr(VI) in 72 h under parthenogenetic anaerobic conditions. In this paper, the complete genome sequence was obtained by Nanopore sequencing technology and analyzed chromium metabolism-related genes by comparative genomics The genomic sequence of *S. putrefaciens* 4H has a length of 4,631,110 bp with a G + C content of 44.66% and contains 4015 protein-coding genes and 3223,  2414, 2343 genes were correspondingly annotated into the COG, KEGG, and GO databases. The qRT-PCR analysis showed that the expression of *chrA*, *mtrC*, and *undA* genes was up-regulated under Cr(VI) stress. This study explores the Chromium Metabolism-Related Genes of *S. putrefaciens* 4H and will help to deepen our understanding of the mechanisms of Cr(VI) tolerance and reduction in this strain, thus contributing to the better application of *S. putrefaciens* 4H in the field of remediation of chromium-contaminated environments.

**Supplementary Information:**

The online version contains supplementary material available at 10.1186/s12864-024-10031-9.

## Introduction

Chromium (Cr) is naturally occurring in almost all environments of the universe including plants, animals, water bodies, soils, rocks, and volcanic gases [1]. With the development of human industry, chromium is widely used in the leather industry [2], metal plating [3], dye processing [4], ink production [5] and other industries, however, this extensive use has also led to the generation of chromium pollution, it is estimated that about 300,000 tons of chromium is released into the environment every year [6], and continuous exposure to chromium causes a variety of hazards to human health. The only two major oxidation states of Cr i.e. [Cr(VI)] and [Cr(III)], exist in water bodies [7]. However, Cr(VI) and Cr(III) differ significantly from one another. The Cr(VI) is extremely toxic to humans and causes cellular carcinogenesis, malformations, DNA damage and hereditary diseases [8], due to its significant toxicity, chromium is classified by the International Agency for Research on Cancer (IARC) as a Group 1 carcinogenic heavy metal [9]. but animals and humans need trace amounts of Cr(III) to sustain a healthy glucose metabolism. It has been documented that Cr(VI) is 100–1000 times more devastating than Cr(III) [10]. Therefore, converting free and extremely poisonous Cr(VI) to stable and minimally toxic Cr(III) is a successful detoxification technique. Currently, the removal of Cr(VI) by ordinary physicochemical techniques is very expensive and not suitable for the removal of low concentrations of Cr. However, the removal and immobilization of Cr(VI) from wastewater using microbial reduction techniques is thought to be a potential approach for remediation of environments polluted with heavy metal because of advantages of low cost and sustainability [11].

*Shewanella* species belong to the Proteobacteria phylum's Gammaproteobacteria class. They have been discovered in sediments and fresh or salt water all around the planet [12]. Due to the versatility of the respiratory system and its ability to dissimilatory metabolism of a broad variety of chemicals such as poisonous elements and variable valence metals, the genus has a high bioremediation potential [13]. It has been reported that Shewanella can not only be suggested as a possible bioremediation agent for anthraquinone and azo dyes [14], but also has a significant impact on the conversion of chromium, arsenic and other metals with variable valence [15, 16]. Among them, the strain named *Shewanella oneidensis* MR-1 has been widely studied, which has been found to have developed multiple mechanisms of resistance to Cr(VI) toxicity, including SOS-controlled DNA repair mechanisms, detoxification, oxidative stress protection, and has become the strain of reference in many researches [17]. Despite the fact that *S. oneidensis* MR-1 is the model bacteria of the genus, many strains of the same genus have also been extensively studied, because in addition to common characteristics, they also exhibit special and useful properties for biotechnology [18].

The *S. putrefaciens* 4H used in this study was previously by our group isolated from the activated sludge of secondary sedimentation tank in dyeing wastewater treatment plant. Considering that *S. putrefaciens* 4H can effectively decompose organic dyes in the previous study of the research group [19], and also has a good effect on the reduction of Cr(VI) in this study. Therefore we optimized the conditions for the reduction of Cr(VI) by *S. put*refaciens 4H in this paper and presented the genome of *S. putrefaciens* 4H, with special emphasis on genes related to chromium metabolism. These data will help to deepen our understanding of the mechanisms of chromium resistance and reduction in this strain, thus contributing to a better application of *S. putrefaciens* 4H in the field of remediation of chromium-contaminated environments.

## Materials and methods

### Morphological characterization of strains, medium and growth conditions

*S. putrefaciens* 4H isolated from the activated sludge of secondary sedimentation tank in dyeing wastewater treatment plant was used in the present study.* S. putrefaciens* 4H was preserved at -80 ℃ in Luria Bertani (LB) liquid culture medium (50%). For microbial activation, 100 µl of bacterial solutions were inoculated into 5 mL of sterilized LB medium containing (/L) 10.0 g peptone, 10 g NaCl, and 5.0 g yeast paste powder (pH 7.0 ± 0.2). Activation cultures were then incubated for 24 h at 37 ℃. After that, 2 mL cultures were added to 100 ml of LB medium to promote bacterial growth. Following a 24-h enrichment period, bacterial liquids were moved to LB liquid culture medium with 100 mg/L Cr(VI) for domestication and enrichment. Repeat the above steps to transfer the bacteria into LB medium containing higher concentration of Cr(VI) for domestication until the bacteria can tolerate 500 mg/L Cr(VI). The domesticated bacteria were transferred to LB medium at 100 mg/L Cr(VI) to maintain their tolerance to Cr(VI) for subsequent use.The bacteria were subjected to Gram staining and microscopic observation of bacterial morphology. Based on the results of the Gram stain, a contact enzyme test is performed if the result is positive, and an oxidase test is performed if it is negative. Then, based on the results of the contact enzyme test or oxidase test, the appropriate card is selected for identification using the VITEK-AMS Fully Automated Bacterial Identification System and the system results were supplemented by biochemical experiments such as nitrate, catalase, IMVIC. The source of Cr(VI) in this investigation was K_2_CrO_4_. Determination of Cr(VI) concentration by diphenylcarbazide (DPC) spectrophotometry at 540 nm [20].

### Environmental parameter optimization for reduction of Cr(VI)

We evaluated the following environmental parameters affecting Cr(VI) reduction by *S. putrefaciens* 4H: temperature (25, 30, 37, 42 ℃), pH (5.0, 6.0, 7.0, 8.0, 9.0, 10.0), initial inoculum (1, 2, 4, 8%), and initial Cr(VI) concentration (0, 100, 300, 400, 500 mg/L) to determine the optimal conditions for the reduction of Cr(VI) by strain 4H in modified LB broth [21]. Cr(VI) reduction by sterile LB medium was served as a blank control. To ensure the accuracy of the experimental data, each experiment was repeated three times.

### Genome sequencing and assembly

Genome sequencing of *S. putrefaciens* 4H was performed at the Wuhan bioyigene Biotechnology Co., Ltd. The extracts were first analyzed by NanoDrop One spectrophotometer (NanoDrop Technologies, Wilmington, DE) and Qubit 3.0 Fluorometer (Life Technologies, Carlsbad, CA, USA) to detect the extracted DNA. When the DNA samples passed the quality control, the DNA was subjected to damage repair and end repair. The barcode label was ligated to the end of the DNA purified by magnetic beads, which was then purified by magnetic beads and ligated to the sequence adapter and created a DNA library. After building the library, the DNA library was sequenced using PromethION (OxfordNanopore Technologies, Oxford, UK) for real-time single-molecule sequencing [22].

Clean reads were obtained after removing the reads with filter adapters, low-quality and short fragments (length < 2000 bp) from the original data. Unicycler (version 0.4.8) [23], pilon (version 1.24) [24], nextpolish (version 1.4.20) [25], circlator (version 1.5.5) [26] were used to combine the data for correction and obtain the final genome sequence.

### Genome annotation and analysis

The coding genes were predicted using prodial and retaining the complete CDS, tRNAs were predicted using tRNAscan-SE [27], rRNA genes were predicted using RNAmmer, other ncRNAs were predicted using infernal by searching the Rfam database [28], CRISPR was predicted using minced, gene islands were predicted using IslandPath-DIMOB, phage prediction with PhiSpy, and repeat number prediction using trf and RepeatMasker [27]. The genomic circle map of the strain was produced using Circos software (http://circos.ca) in accordance with the fundamental genome characteristics and the findings of the bioinformatics investigation based on integration of GC ratio, GC-skew, and Genome sequencing depth.

The predicted coding protein sequences in *S. putrefaciens* 4H were compared and functionally annotated with the coding protein sequences from the COG, KEGG, GO, Refseq, Pfam, SwissProt and TIGRFAMs databases, and categorize the protein functions annotated to COG, KEGG and GO databases [29].

### Prediction of Secondary metabolic

AntiSMASH [30] was utilized for the prediction of gene clusters of secondary metabolites of *S. putrefaciens* 4H by a Hidden Markov Model (HMM) based on the gene information of some specific types of gene clusters.

### Comparative genome analysis of chromium metabolism-related genes

Whole genome sequence comparison was performed using the TrueBac™ ID system in EzBioCloud (https://www.ezbiocloud.net/) [31], from which the 12 model strains with the highest similarity to the 4H genome sequence were selected and their whole genome sequences were downloaded. The average nucleotide identity (ANI) and DNA-DNA hybridization (DDH) values between the above genomes were calculated using the JSpeciseWS (https://jspecies.ribohost.com/jspeciesws/) and GGDC (https://ggdc.dsmz.de/) platforms values (DDH) [32].

To further investigate the genomic characterization of strain 4H, four of the 12 model strains with the highest genomic sequence similarity to strain 4H were selected for comparative genomics analysis. Gene collinearity analysis was performed using Mauve software [33]. Core genome and pan-genome analyses were performed using core genes and specific genes of the five strains extracted using the Orthofinder software package. The CDS sequences of core genes in Orthofinder were annotated using KEGG Mapper (https://www.kegg.jp/kegg/mapper/assign_ko.html) [34] and eggNOG-mapper (http://eggnog-mapper.embl.de/) [35]. Proteins related to chromium metabolism were analyzed based on whole genome sequencing as well as comparative genomics annotation [36].

### Quantitative real time PCR

Strain 4H was cultured in LB medium until mid-exponential growth, and 2% of the inoculum was added to LB medium with Cr(VI) concentrations of 0, 300, and 500 mg/L, respectively, and grown under chromium stress at 30 ℃ and 120 rpm for 24 h. Bacteria were collected by centrifugation 5 min at 8000 r/min at 4 ℃ and washed twice with phosphate buffer (pH 7.4). The sample was sent to Wuhan bioyigene Biotechnology Co., Ltd. for quantitative real-time PCR. qRT-PCR was carried out on Real-Time PCR System (Applied Biosystems StepOnePlus™) at the following procedures: 1 cycle of 95 °C for 30 s, 40 cycles of 95 °C for 10 s, 60 °C for 30 s. Primer specificity was verified prior to formal experiments. Primers used in qRT-PCR were listed in Table S2. 16S rRNA gene was selected to normalize the data, and qRT-PCR data were analyzed using the 2^−ΔΔCt^ method [37].

## Results

### Bacterial morphology and characteristics

The liquid culture of the bacteria had a characteristic odor slightly lighter than that of rotten eggs. The bacterium appeared as rod-shaped under microscopic observation and the color of colonies ranged from beige to pink according to their different growth stages on LB agar plates, and the colony was round shape with smooth surface (Fig. S1). Bacterial Gram stain results were negative, therefore an oxidase test was performed on it. The result of the oxidase test was positive and the bacterium was judged to be a non-fermenting bacterium. The pure strain was incubated at 50 degrees for 6 h and the GNI drug sensitivity card of the non-fermenting bacteria was selected for fully automated identification, which revealed that *S. putrefaciens* 4H is positive for maltose and sucrose and produces H_2_S, which is the source of its characteristic odor, in addition to the metabolic process that may also produce ornithine. At the same time, physiological and biochemical experiments demonstrated that the bacterium contains catalase as well as nitrate reductase (Table 1). Through long-term domestication, the strain was capable to grow in 500 mg/L Cr(VI).

**Table 1:** Physiological and biochemical characterization of *S. putrefaciens* 4H

Testing Program		Result	Testing Program		Result
DP3	DP-300	-	SOR	Sorbitol	-
OFG	Glucose(oxidative)	-	SUC	Sucrose	+
GC	Growth Control	+	INO	Inositol	-
ACE	Acetamide	-	ADO	Adonitol	-
ESC	Escμlin	-	COU	p-Coumaric	-
PLI	Plant Indican	-	H_2_S	H_2_S	+
URE	Urea	-	ONP	ONPG	-
CIT	Citrate	-	RHA	Rhamnose	-
MAL	Malonate	-	ARA	Arabinose	-
TDA	TDA	-	GLU	Glucose(Fermentative)	-
PXB	Polymyxin B	-	ARG	Arginine	-
LAC	Lactose	-	LYS	Lysine	-
mlT	Maltose	+	ORN	Ornithine	+
MAN	Mannitol	-	OXI	Oxidase	+
XYL	Xylose	-	TLA	10%lactose	-
RAF	Raffinose	-	NIT	Nitrate	+
CAT	Catalase	+	TRP	tryptophanase	-

+ indicates a positive reaction, -indicates a negative reaction

### Impact of various factors on Cr(VI) reduction

Optimization of environmental parameters is an important prerequisite for the study of microbial reduction of Cr(VI), therefore, it is important to evaluate the effect of environmental parameters on the reduction of Cr(VI).

#### pH effect on Cr(VI) remediation

**Fig. 1:**
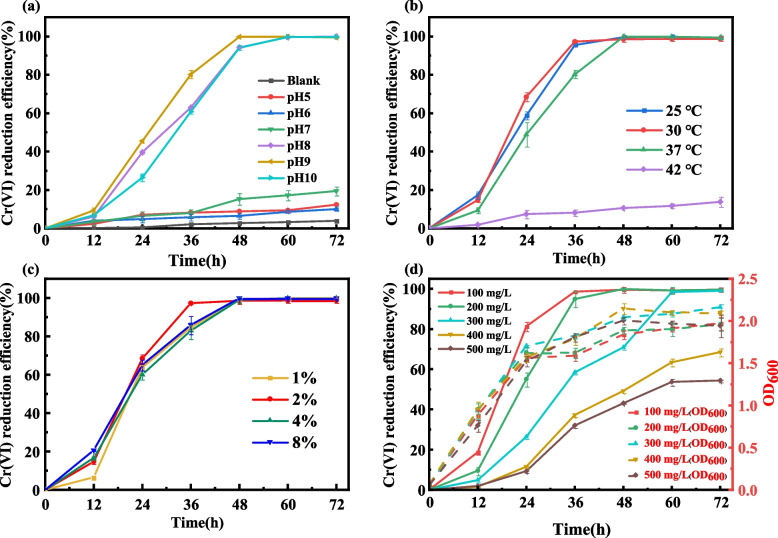
Effect of different parameters on Cr(VI) reduction. **a** Effect of pH. **b** Effect of temperature. **c** Effect of inoculation volume. **d** Effect of initial Cr(VI) concentration. The data are presented as the mean (*n* = 3) ± standard deviation (S.D)

From the Fig. 1a, it can be seen that *S. putrefaciens* 4H has the ability to reduce Cr(VI) under the condition of pH 5–10, but the reduction rate at pH 8, 9 and 10 is much higher than that at pH 5, 6 and 7, and complete reduction was achieved at initial Cr (VI) concentrations between 100 and 300 mg/L after 72 h. The above results indicated that *S. putrefaciens* 4H was effective in reducing Cr(VI) under neutral to alkaline conditions and the optimum pH was 9, which may be due to the fact that the optimal pH for the growth of this genus of bacteria is alkaline, and suitable pH conditions are favorable for the proliferation of the bacteria [38]. The low reduction rate under acidic conditions may be due to the fact that the low pH affects the integrity of cell membrane, permeability and ion channels, which prevented the normal growth and metabolism of the bacterium, resulting in a significant reduction in the reduction rate of Cr(VI) [39].

#### Effect of temperature

The strain is capable of reducing Cr(VI) in a wide range of temperatures from 25 ℃ to 37 ℃, with the optimum temperature being 30 ℃ (Fig. 1b). As can be seen, the strain is well adapted to temperature, but the reduction efficiency of the strain will be reduced if the temperature is too high, which may be due to the growth of *S. putrefaciens* 4H is inhibited by the high temperature [40].

#### Effect of inoculation volume

The inoculation volume also greatly affected the Cr(VI) reduction rate (Fig. 1c). With higher the inoculation amount, the faster the reduction rate of Cr(VI) was achieved at the early stage of bacterial growth, and the sufficient quantity of bacteria provided the acclimation period for bacteria to adjust to chromium stress environments more quickly.

#### Influence of initial Cr(VI) concentration

The strain could almost complete the reduction of Cr(VI) within 72 h when the initial concentration of Cr(VI) was between 100 ~ 300 mg/L (Fig. 1d). After 72 h, 71% of Cr(VI) at an starting concentration of 400 mg/L was reduced, while at starting concentration of 500 mg/L, 53% of Cr(VI) was reduced after 72 h. The reduction rate of Cr(VI) at this time was basically unchanged compared to that at 60 h, indicating that the reduction of Cr(VI) by the bacterium in the experimental environment basically reached the threshold and the existing conditions could not completely reduce the remaining Cr(VI). Apparently the strains were effective in reducing Cr(VI) from 0 ~ 500 mg/L (w/v), and the reduction efficiency was slower at high initial Cr(VI) concentrations, which may be due to the toxicity of Cr(VI) affecting the growth of the strains [37].

### Genome properties and analysis

By performing gene assembly and structural annotation on the sequencing data after quality control, the genome was characterized as in Table S1. The genome of *S. putrefaciens* 4H has a length of 4,631,110 bp with the GC content of 44.66%. A total of 6469 genes were predicted and of those 4015 were coding sequences (CDS), 250 were pseudo genes and 132 were tRNAs/rRNAs genes. The genome data of *S. putrefaciens* 4H was submitted to NCBI with an accession number CP104755.1, Bio-Project and Bio-Sample accession numbers were designated as PRJNA881029 and SAMN30884599, respectively. Circos online mapping of the nuclear genome circle shows nuclear genome sequencing depth, GC distribution, GC-skew, and genome structure, with more information shown in Fig. S2.

### Functional gene annotation

#### COG database annotation

**Fig. 2:**
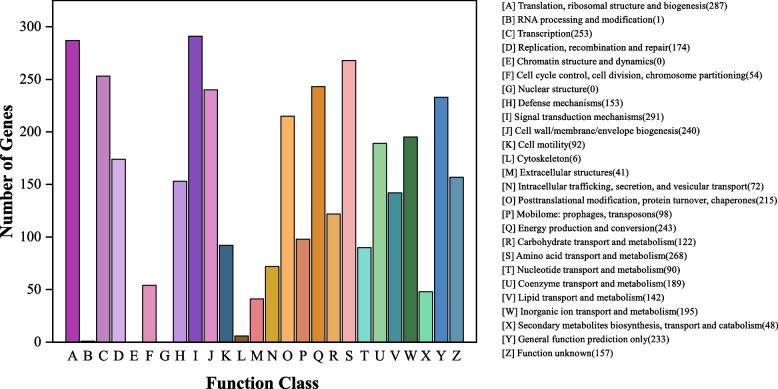
COG function classification of genes in *S. putrefaciens* 4H

To categorize the homologous annotations, the COG database was used to algin all the predicted CDS sequences of *S. putrefaciens* 4H. Overall, 3223 protein coding sequences were effectively annotated into COG (Fig. 2), where they were divided into 26 categories ranging from A to Z. In which the greatest number of genes belonged to the family of “signal transduction mechanisms” (291) followed by “translation, ribosomal structure and biogenesis” (287), “amino acid transport and metabolism” (268) and “Transcription” (253). Meanwhile, “the inorganic ion transporter” genes (195) and the “DNA replication, recombination, and repair” genes (174) were also present in a certain proportion, which inferred that these genes could be crucial for bacteria’s absorption and outflow of Cr(VI) and resist the toxic effects of Cr(VI) by self-repairing the damaged DNA [41].

#### KEGG database annotation

**Fig. 3:**
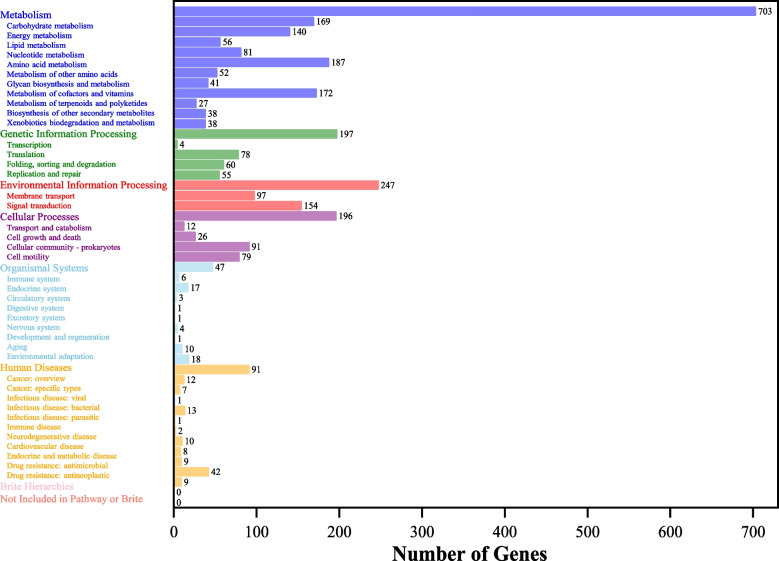
KEGG pathway classification of genes in *S. putrefaciens* 4H

A total of 4015 protein coding sequences were functionally annotated using the KEGG database, of which 2414 protein coding sequences were categorized and counted into eight major classes, each with several subclasses (Fig. 3). The most of them fall into one of the following categories: 187 protein sequences in amino acid metabolism, 172 in metabolism cofactors vitamins, 169 in carbohydrate metabolism 154 in signal transport, 140 in energy metabolism, 97 in membrane transport, and 81 in nucleotide metabolism. It is worth noting that the *S. putrefaciens* 4H genome accounted for a sizable portion of the genes involved in substance transport and metabolism, energy metabolism, and membrane transport in the annotated results of the KEGG, which indicates that the bacterium has a strong ability to metabolize substances. Therefore, when Cr(VI) gets inside the cell, the stress response it produces may involve membrane transport and substance transport metabolism, in which a large number of transporters and channel proteins may play key roles.

#### GO database annotation

**Fig. 4:**
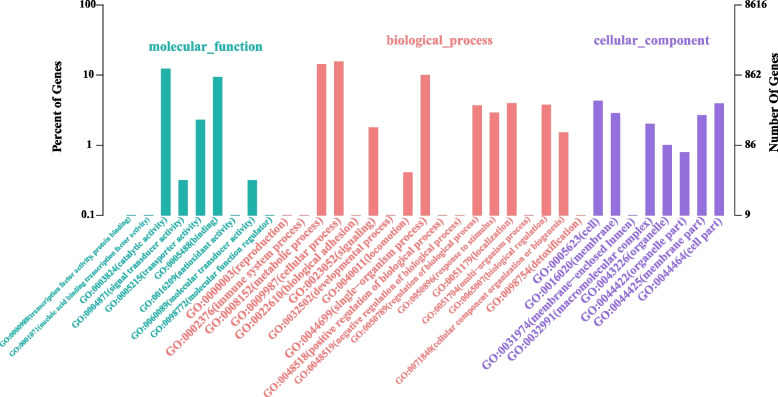
The GO function classification chart of *S. putrefaciens* strain 4H functional annotation

There were 2343 GO annotation sequences for *S. putrefaciens* 4H, which were grouped into three main categories with a total of 35 sub-functions, according to the GO functional annotation results, the genes involved in membrane, macromolecular complexes, and cell accounted for a significant share of the cellular components (Fig. 4). In the category of biological process, the majority sequences were connected to cellular processes and metabolic processes, further suggesting that *S. putrefaciens* 4H may complete the Cr(VI) metabolic process via intracellular activities. The primary annotated sequences in the area of molecular unction category were catalytic activities, transport activities, and binding, and these are related to the activity of cells and the ability to transport substances, indicating that the *S. putrefaciens* 4H has excellent replication and material metabolic functions.

### Predictive analysis of secondary metabolic

**Fig. 5:**
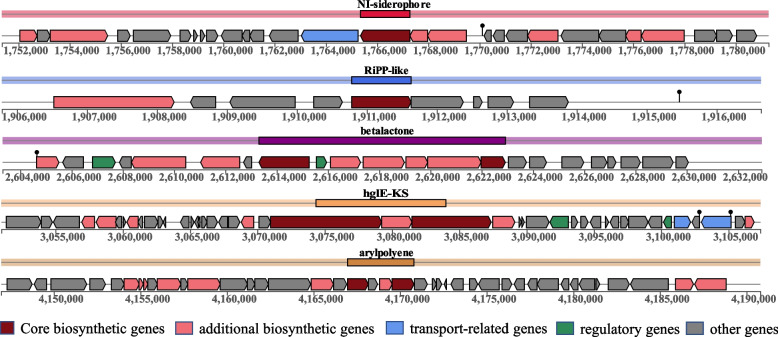
Secondary metabolite synthesis clusters predicted by antiSMASH

AntiSMASH predicted a total of five gene clusters for secondary metabolite synthesis: siderophore, RiPP-like, beta lactone, PUFA, hglE-KS and aryl polyene. These groups of genes included genes involved in additional biosynthetic, core biosynthetic, transport-related, regulatory and other genes (Fig. 5), in which siderophore have been extensively studied. Siderophores are the most effective way for microorganisms to take up iron from iron-poor environments [42], bacteria containing siderophores use specific ATP-dependent membrane-associated transporters deliver the Fe(III)–siderophore complex to the cell [43]. Inside the cell, Fe(III) is enzymatically reduced to soluble and solid-phase Fe(II), which quickly transfers its electron to Cr(VI), becoming the reductants for Cr(VI) [44]. Therefore, the presence of ferric carrier gene clusters may be related to the transfer of Fe valence state in the cells of *S. putrefaciens* 4H, which directly or indirectly affects the reduction of Cr(VI) by the strain.

### Comparative genome analysis of chromium metabolism-related genes

The phylogenetic tree was constructed using the TYGS platform as shown in Fig. S3, the closer evolutionary distance between *S. putrefaciens* 4H and *S. putrefaciens* CN-32 suggests that the two may possess a closer kinship, which may be manifested in ecological adaptation, and that the two may survive under similar environmental conditions and have similar biological characteristics. ANI (Average Nucleotide Identity) DDH (DNA-DNA hybridization) as shown in Fig. S4 and Table S3. and the results showed that the strains *S. putrefaciens* 4H and *S. putrefaciens* CN-32 had ANI and DDH values as high as 98.33% and 87.3%. The similarity between the genomes of *S. putrefaciens* 4H and *S. putrefaciens* CN-32 was further demonstrated [45], providing clues and directions for in-depth genomic analysis to identify candidate genes related to chromium resistance and reduction. The gene collinearity analysis of the above strains was analyzed using Mauve software, and the strong syntenic relationships were found between *S. putrefaciens* 4H and *S. putrefaciens* CN-32 (Fig. S5). However, there were various degrees of gene rearrangements such as insertions, deletions, translocations and inversions between the genomes of *S. putrefaciens* 4H and the control strain. These gene rearrangements may be the result of genetic changes that may be influenced by factors such as environmental stress, adaptive selection and gene transfer.

The CDS sequences of the above five genomes were clustered and analyzed for orthologous genes using Orthofinder, and a total of 4,302 orthologous genes were obtained (Fig. S6), of which 2,790 were core genes, accounting for 64.85% of the total orthologous genes. The KEGG and COG database annotations for the core genes are shown below Figs. S7 and S8. The genes related to "metabolism" accounted for the largest proportion in the core genome. The results are consistent with the previous analysis of *S. putrefaciens* 4H genome, which demonstrated that *S. putrefaciens* 4H and several other strains with similar affinity have strong metabolic ability.

By homology comparison of databases, we predicted the presence of chromium resistance-related genes *chrA* in the genome sequence, whose expressed ChrA was a membrane protein that resists the chromate toxicity by expelling intracellular chromate ions [46]. ChrA has been identified in *Shewanella* sp. ANA-3 [47], *S. oneidensis* MR-1 [48], *Shewanella putrefaciens* CN-32 of the same genus, of which *S. putrefaciens* CN-32 is high homology to *S. putrefaciens* 4H. Therefore, it is speculated that ChrA may play the same role in *S. putrefaciens* 4H.

In addition to the efflux of Cr(VI), reduction of Cr(VI) to Cr(III) by reductase is another effective way to reduce cellular toxicity. Quinone reductases, hydrogenases, iron reductase, nitroreductase, NAD(P)H-dependent reductases and flavin reductases are among the six main types of Cr(VI) reductases that have so far been found [49]. We found that the possible genes associated with chromium reduction included nitroreductase *nfsB*, azo reductase of *azoR* (Table 2). Furthermore, it has also been reported that Cr(VI) can act as an extracellular electron transfer receptor in *S. oneidensis* MR-1 [50]. Therefore, homology alignment of cytochrome *c* proteins diversity in the sequenced *S. putrefaciens* 4H genomes by using NCBI revealed that *S. putrefaciens* 4H encoded respiratory system that is similar to those identified in *S. putrefaciens* CN-32 and *S. putrefaciens* 200, including those that involve cell-surface-localized electron transfer proteins, which enable their hosts have the capacity to mediate direct electron transfer to outer membrane electron acceptors, including cytochrome *c* proteins UndA, MtrA, MtrB, and MtrC. The majority of the aforementioned genes have been discovered in the genome of *S. putrefaciens* 4H, proving that these genes are crucial for *S. putrefaciens* 4H survival under high Cr(VI) concentrations.

**Table 2:** Genes related to chromium metabolism in *S. putrefaciens* 4H genome

Locus tag	Function	Gene name	Degree of homology
N5094_04120	Chromate transporter	*chrA*	*S. putrefaciens* CN-32 (100%)
N5094_12900	11-heme c-type cytochrome	*undA*	*S. putrefaciens* 200 (99.92%)
N5094_12905	periplasmic decaheme cytochrome c	*mtrC*	*S. putrefaciens* 200 (99.64%)
N5094_12910	cytochrome C family protein	*mtrA*	*S. putrefaciens* CN-32 (97.52%)
N5094_12915	outer membrane protein	*mtrB*	*S. putrefaciens* 200 (99.62%)
N5094_18465	FMN-dependent NADH-azo reductase	*azoR*	*Shewanella baltica* OS678 (93.47%)
N5094_04225	NAD(P)H-dependent nitroreductase	*nfsB*	*S. putrefaciens* CN-32 (98.47%)

### Analysis of qRT-PCR results

**Fig. 6:**
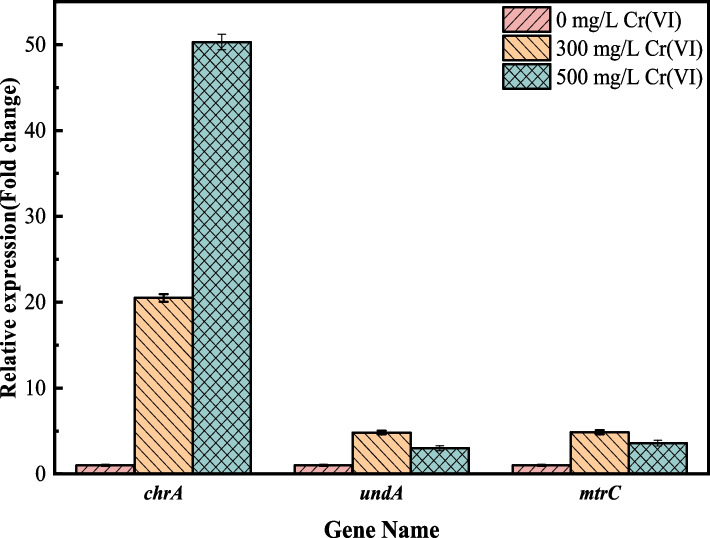
Relative fluorescence quantitative PCR results. The data are presented as the mean (*n* = 3) ± standard deviation (S.D), error bars represent S.D

To further verify the expression of the above genes under chromium stress environment, we selected *chrA*, *mtrC*, *undA* for fluorescence quantification experiments. The results of relative fluorescence quantitative PCR are shown in Fig. 6. The expression of *chrA*, *undA* and *mtrC* genes were induced by Cr(VI), i.e., the transcript levels of the genes increased with the initial Cr(VI) concentration. Compared with the control group, i.e., no Cr(VI) stress, the expression level of *chrA* gene was up-regulated 20.49-fold when the Cr(VI) stress concentration was 300 mg/L, and 50.3-fold when the Cr(VI) stress concentration was 500 mg/L. The chrA gene has been shown to be associated with Cr(VI) efflux in several studies [48], and its efflux capacity was significantly enhanced with the increase of Cr(VI) concentration, suggesting that the bacterium reduces intracellular Cr(VI) content mainly by means of efflux and thus resists the toxicity of high concentrations of Cr(VI) to the cells.

When the Cr(VI) stress concentration was 300 mg/L, the expression levels of *undA* and *mtrC* genes were up-regulated by 4.83- and 4.85-fold, respectively, compared with the control group, and many studies have shown that these two genes, as the terminal reductase of extracellular electron transfer cytochrome *c* in *S. putrefaciens*, are importantly related to the metabolism of microorganisms in the reduction of heavy metals. However, when the Cr(VI) stress concentration was 500 mg/L, the expression level was slightly decreased compared with that of 300 mg/L, which might be due to the toxic effect of high concentration of Cr(VI) on the bacteria, affecting their own ability to metabolize heavy metals, which led to the decrease in the expression level.

## Discussion

This study discovered that *S. putrefaciens* 4H has the ability to reduce chromate and exhibit high resistance to chromate. To further investigate its genomic characteristics and chromium metabolism-related genes, we performed whole genome sequencing and gene comparative genomics analysis. The study found that the genome of facultative *S. putrefaciens* 4H is rich in genes associated with inorganic ion transport as well as metabolic functions. The phylogenetic tree constructed based on the whole genome, as well as DDH and ANI values, indicate a close relationship between this bacterium and *S. putrefaciens* CN-32. By analyzing and comparing their core gene annotations, it was predicted that Cr-tolerance-related proteins ChrA and Cr-reduction-related functional genes azo reductase AzoR, nitroreductase NfsB, and cytochrome *c* proteins UndA, MtrC, MtrA, MtrB may exist in the genome of *S. putrefaciens* 4H.

The *chrA* gene encodes transporters associated with chromium resistance, which has been documented to be involved in Cr(III) and Cr(VI) transport through various roles such as transmembrane proton gradient, generating membrane potential, and electron transfer [51]. When bacteria are exposed to chromium stress, Cr(VI) enters cells through sulfate ion channels and is carried out of cells by ChrA transmembrane proteins, thereby reducing the damage caused by hexavalent chromium. In order to study the gene expression of *chrA*, researchers have transformed the *chrABC* gene from *Shewanella* sp. ANA-3 into *Escherichia coli*. The findings demonstrated that the resistance to chromium with *chrA* gene was boosted, and the mechanism of chromium resistance is likely to be accomplished by chromium efflux [47]. Since the *chrA* gene in this study had 100% homology with that in *S. putrefaciens* CN-32, it is hypothesized that they may have theoretically similar anti-chromium mechanisms. The results of qRT-PCR also demonstrated that as the chromium concentration increased, the expression level of this gene significantly increased.

Some nitroreductase and azo reductase genes were found in *S. putrefaciens* 4H, of which Nitroreductase NfsB and azo reductase AzoR have been documented in the research of *Escherichia coli* [52] and *Vibrio harveyi* KCTC 2720 [53]. Even though these genes are not particular for reducing Cr(VI), these reductases could still be involved. In addition to enzymes, another method is the reduction of Cr(VI) through its special electron transport pathway, most of the current studies are based on the model strain *S. oneidensis* MR-1 of the genus Shewanella. Under anaerobic conditions, *S. oneidensis* MR-1 can reduce a different kinds of compounds, including fumarate, nitrate, manganese and iron oxides [54], and this is because of its diverse electron transfer pathways. The special pathways give *S. oneidensis* MR-1 an extraordinary ability to transform substances and putrefaciens and is known collectively by researchers as the Mtr pathway (Metal Reduction Pathway), including CymA, MtrA, OmcA, MtrC, and other cytochrome c as well as MtrB, which connects periplasmic space with outer membrane proteins [55]. According to previous reports, MtrA is a periplasmic 10-haem *c*-type cytochrome that seems to be component of the electron transport chain that results in the reduction of Fe(III), and MtrC is an outer membrane 10-haem *c*-type cytochrome [56] that works in conjunction with OmcA to transfer electrons to extracellular [57]. In the strain *S. oneidensis* MR-1, extracellular Cr(VI) can be reduced by receiving electrons transferred by this pathway as the terminal electron acceptor, where MtrC and OmcA are the terminal reductases used for extracellular Cr(VI) reduction [50], and deletion of the MtrC and OmcA may lead to the decreased of Cr(VI) reduction efficiency on the outer membrane [58].

**Fig. 7:**
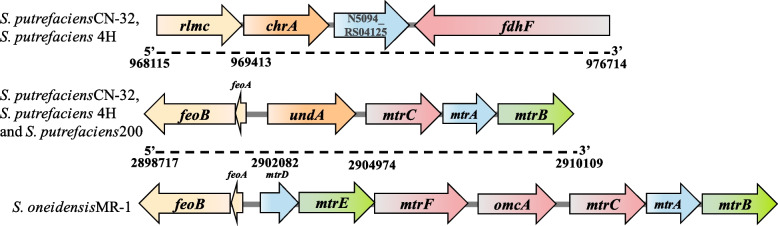
Prediction of the chromium metabolism gene cluster in *S. putrefaciens* 4H

By comparison, it was found that *S. putrefaciens* 4H and *S. oneidensis* MR-1 all exist a single locus that encodes a 10-haem cytochrome *c* that is present in the periplasm (MtrA) and on the surface of cells (MtrC), as well as the outer-membrane protein (MtrB) that is necessary for the reduction of metal oxides but whose function is unknown (Fig. 7) [13]. However, different from *S. oneidensis* MR-1, *S. putrefaciens* 4H has 11-hame(*undA*) *c*-type cytochromes in its gene cluster, while the *S. oneidensis* MR-1 has 10-haem(*omcA* and *mtrF*) *c*-type cytochromes and carries the similarly related but not proved *mtrDEF* genes in this region, all of which are projected to be confined to the outer membrane. The qRT-PCR results showed that *undA* as well as *mtrC* were expressed in the chromium environment. However, the majority of these extra genes are regulated by distinct promoters, indicating that each protein can be expressed in response to different signals while also reflecting differences in substrate specificity [13]. Therefore, whether the above genes are specific for Cr(VI) is unknown, and the functions and specific expression remain to be further verified.

## Conclusions

In this study, we optimized the environmental parameters for Cr(VI) reduction by microbial strains and assembled the whole genome of *S. putrefaciens* 4H. Comparative genome analysis showed that the genomes of this bacterium and *S. putrefaciens* CN-32 exhibited strong syntenic relationships, and had a strong substance metabolizing ability. In addition, they share similar clusters of electron transport genes, which differ from the model strain of the same genus, *S. oneidensis* MR-1. The presence of Cr(VI) tolerance-related protein (ChrA) gene and Cr(VI) reduction-related protein (MtrC, UndA, MtrB, MtrC, AzoR, NfsB) genes was predicted in *S. putrefaciens* 4H by the above analysis. The expression of *chrA*, *mtrC*, and *undA* under heavy metal stress was further investigated by qRT-PCR.

Collectively, the information from genome sequence is helpful for identifying potential heavy metal resistance genes in *S. putrefaciens* 4H and sheds light on the adaptability and versatility of this strain. It also provides a theoretical framework for the use of *S. putrefaciens* as well as genetic engineering techniques to improve chromium-contaminated environments.

### Supplementary Information

**Additional file 1:** **Fig. S1.** Morphology and Gram staining of *S. putrefaciens* 4H colonies(1500x). **Fig. S2.** Nuclear genome circle diagram. From outside to inside, coding genes (righteous strand), coding genes (negative-sense strand), tRNA (orange) and rRNA (purple), CRISPR, prophage and gene islands, GC ratio, GC-skew, sequencing depth. **Fig. S3.** Whole genome sequence phylogenetic tree. **Fig. S4.** Average nucleotide identity (ANI) values of *S. putrefaciens* 4H with similar model strains. **Fig. S5.** Gene collinearity analysis of * S. putrefaciens *4H and four closely related model strains. **Fig. S6.** Venn diagram of direct homologous gene between *S. putrefaciens* 4H and four closely related model strains. **Fig. S7**. KEGG functional annotation analysis of the core genes. **Fig. S8.** Functional annotation analysis of COG core genes.  **Table S1.** General features of *S. putrefaciens* 4H. **Table S2.** Primers for RT-qPCR experiments. **Table S3.** DDH values of *S. putrefaciens* 4H and similar model strains.

## Data Availability

The data presented in this study are openly available in the National Center for Biotechnology Information (https://www.ncbi.nlm.nih.gov/nuccore/CP104755.1/).

## References

[1] Joutey NT, Sayel H, Bahafid W, El Ghachtouli N (2015). Mechanisms of hexavalent chromium resistance and removal by microorganisms. Rev Environ Contam Toxicol.

[2] Jimenez-Paz J, Lozada-Castro JJ, Lester E, Williams O, Stevens L, Barraza-Burgos J (2023). Solutions to hazardous wastes issues in the leather industry: adsorption of Chromium iii and vi from leather industry wastewaters using activated carbons produced from leather industry solid wastes. J Environ Chem Eng.

[3] Woo S-Y, Kim J-S, Woo J-H, Oh S-U, Kim Y-D (2023). Feasibility assessment of humidification–dehumidification and adsorption water treatment processes for real-time treatment of plating industry wastewater. Chem Eng J.

[4] Liu Z, Khan TA, Islam MA, Tabrez U (2022). A review on the treatment of dyes in printing and dyeing wastewater by plant biomass carbon. Bioresour Technol.

[5] Zampeta C, Paparouni C, Tampakopoulos A, Frontistis Z, Charalampous N, Dailianis S, Koutsoukos PG, Paraskeva CA, Vayenas DV (2022). Printing ink wastewater treatment using hydrodynamic cavitation and coagulants/flocculants. J Environ Manag.

[6] Ifthikar J, Shahib II, Jiang W, Senthilnithy R, Elkhlifi Z, Wang J, Chen Z (2023). Review on technologies for the development of effective and practical chromate removal from wastewaters. J Environ Chem Eng.

[7] Cespón-Romero RM, Yebra-Biurrun MC, Bermejo-Barrera MP (1996). Preconcentration and speciation of chromium by the determination of total chromium and chromium(III) in natural waters by flame atomic absorption spectrometry with a chelating ion-exchange flow injection system. Anal Chim Acta.

[8] Bai Y, Long C, Hu G, Zhou D, Gao X, Chen Z, Wang T, Yu S, Han Y, Yan L (2019). Association of blood chromium and rare earth elements with the risk of DNA damage in chromate exposed population. Environ Toxicol Pharmacol.

[9] Kim HS, Kim YJ, Seo YR (2015). An overview of carcinogenic heavy metal: molecular toxicity mechanism and prevention. J Cancer Prev.

[10] Miretzky P, Cirelli AF (2010). Cr(VI) and cr(III) removal from aqueous solution by raw and modified lignocellulosic materials: a review. J Hazard Mater.

[11] Tang X, Huang Y, Li Y, Wang L, Pei X, Zhou D, He P, Hughes SS (2021). Study on detoxification and removal mechanisms of hexavalent chromium by microorganisms. Ecotoxicol Environ Saf.

[12] Hau HH, Gralnick JA (2007). Ecology and biotechnology of the genus Shewanella. Annu Rev Microbiol.

[13] Fredrickson JK, Romine MF, Beliaev AS, Auchtung JM, Driscoll ME, Gardner TS, Nealson KH, Osterman AL, Pinchuk G, Reed JL (2008). Towards environmental systems biology of Shewanella. Nat Rev Microbiol.

[14] Liu W, Liu L, Liu C, Hao Y, Yang H, Yuan B, Jiang J (2016). Methylene blue enhances the anaerobic decolorization and detoxication of azo dye by Shewanella onediensis MR-1. Biochem Eng J.

[15] Wang J, Wu M, Lu G, Si Y (2016). Biotransformation and biomethylation of arsenic by Shewanella oneidensis MR-1. Chemosphere.

[16] Sreedevi PR, Suresh K, Jiang G (2022). Bacterial bioremediation of heavy metals in wastewater: a review of processes and applications. J Water Process Eng.

[17] Brown SD, Thompson MR, Verberkmoes NC, Chourey K, Shah M, Zhou J, Hettich RL, Thompson DK (2006). Molecular dynamics of the Shewanella oneidensis response to chromate stress. Mol Cell Proteomics.

[18] Zhong C, Han M, Yu S, Yang P, Li H, Ning K (2018). Pan-genome analyses of 24 Shewanella strains re-emphasize the diversification of their functions yet evolutionary dynamics of metal-reducing pathway. Biotechnol Biofuels.

[19] Jiang Y, Gui Z, Qi HH, Luo AJ, Liu L, Ye JH, Cai YJ (2015). Screening, indentifacation and decoloration study of a Shewanella putrtfaciens strain. J Anhui Agricultural Sci.

[20] Chen GQ, Zhang WJ, Zeng GM, Huang JH, Wang L, Shen GL (2011). Surface-modified Phanerochaete chrysosporium as a biosorbent for cr(VI)-contaminated wastewater. J Hazard Mater.

[21] Lizarraga WC, Mormontoy CG, Calla H, Castaneda M, Taira M, Garcia R, Marin C, Abanto M, Ramirez P (2022). Complete genome sequence of Shewanella algae strain 2NE11, a decolorizing bacterium isolated from industrial effluent in Peru. Biotechnol Rep (Amst).

[22] Senol Cali D, Kim JS, Ghose S, Alkan C, Mutlu O (2019). Nanopore sequencing technology and tools for genome assembly: computational analysis of the current state, bottlenecks and future directions. Brief Bioinform.

[23] Wick RR, Judd LM, Gorrie CL, Holt KE (2017). Unicycler: resolving bacterial genome assemblies from short and long sequencing reads. PLoS Comput Biol.

[24] Walker BJ, Abeel T, Shea T, Priest M, Abouelliel A, Sakthikumar S, Cuomo CA, Zeng Q, Wortman J, Young SK (2014). Pilon: an integrated tool for comprehensive microbial variant detection and genome assembly improvement. PLoS One.

[25] Hu J, Fan J, Sun Z, Liu S (2020). NextPolish: a fast and efficient genome polishing tool for long-read assembly. Bioinformatics.

[26] Hunt M, Silva ND, Otto TD, Parkhill J, Keane JA, Harris SR (2015). Circlator: automated circularization of genome assemblies using long sequencing reads. Genome Biol.

[27] Lowe TM, Chan PP (2016). tRNAscan-SE On-line: integrating search and context for analysis of transfer RNA genes. Nucleic Acids Res.

[28] Nawrocki EP, Burge SW, Bateman A, Daub J, Eberhardt RY, Eddy SR, Floden EW, Gardner PP, Jones TA, Tate J (2015). Rfam 12.0: updates to the RNA families database. Nucleic Acids Res.

[29] Dong L, Zhou S, He Y, Jia Y, Bai Q, Deng P, Gao J, Li Y, Xiao H (2018). Analysis of the genome and chromium metabolism-related genes of Serratia sp. S2. Appl Biochem Biotechnol.

[30] Blin K, Shaw S, Kloosterman AM, Charlop-Powers Z, van Wezel GP, Medema MH, Weber T (2021). antiSMASH 6.0: improving cluster detection and comparison capabilities. Nucleic Acids Res.

[31] Yoon SH, Ha SM, Kwon S, Lim J, Kim Y, Seo H, Chun J (2017). Introducing EzBioCloud: a taxonomically united database of 16S rRNA gene sequences and whole-genome assemblies. Int J Syst Evol Microbiol.

[32] Yan Z, Li M, Wang J, Pan J (2019). Genome analysis revealing the potential mechanisms for the heavy metal resistance of Pseudomonas sp. P11, isolated from industrial wastewater sediment. Curr Microbiol.

[33] Chen YJ, He GC, Cheng JF, Lee YT, Hung YH, Chen WH, Huang YT, Liu PY (2020). Comparative genomics reveals insights into characterization and distribution of quorum sensing-related genes in Shewanella algae from marine environment and clinical sources. Comp Immunol Microbiol Infect Dis.

[34] Kanehisa M (2019). Toward understanding the origin and evolution of cellular organisms. Protein Sci.

[35] Cantalapiedra CP, Hernandez-Plaza A, Letunic I, Bork P, Huerta-Cepas J (2021). eggNOG-mapper v2: functional annotation, orthology assignments, and domain prediction at the metagenomic scale. Mol Biol Evol.

[36] Gomez-Garzon C, Hernandez-Santana A, Dussan J (2016). Comparative genomics reveals Lysinibacillus sphaericus group comprises a novel species. BMC Genomics.

[37] Zhu Y, Yan J, Xia L, Zhang X, Luo L (2019). Mechanisms of cr(VI) reduction by Bacillus sp. CRB-1, a novel cr(VI)-reducing bacterium isolated from tannery activated sludge. Ecotoxicol Environ Saf.

[38] Yang S-P, Xie J, Cheng Y, Zhang Z, Zhao Y, Qian Y-F (2020). Response of Shewanella putrefaciens to low temperature regulated by membrane fluidity and fatty acid metabolism. LWT.

[39] Wu M, Li Y, Li J, Wang Y, Xu H, Zhao Y (2019). Bioreduction of hexavalent chromium using a novel strain CRB-7 immobilized on multiple materials. J Hazard Mater.

[40] An H, Tian T, Wang Z, Jin R, Zhou J (2022). Role of extracellular polymeric substances in the immobilization of hexavalent chromium by Shewanella putrefaciens CN32 unsaturated biofilms. Sci Total Environ.

[41] Viamajala S, Peyton BM, Apel WA, Petersen JN (2002). Chromate/nitrite interactions in Shewanella oneidensis MR-1: evidence for multiple hexavalent chromium [Cr(VI)] reduction mechanisms dependent on physiological growth conditions. Biotechnol Bioeng.

[42] Wilson BR, Bogdan AR, Miyazawa M, Hashimoto K, Tsuji Y (2016). Siderophores in iron metabolism: from mechanism to therapy potential. Trends Mol Med.

[43] Soe CZ, Telfer TJ, Levina A, Lay PA, Codd R (2016). Simultaneous biosynthesis of putrebactin, avaroferrin and bisucaberin by Shewanella putrefaciens and characterisation of complexes with iron(III), molybdenum(VI) or chromium(V). J Inorg Biochem.

[44] Cummings DE, Fendorf S, Singh N, Sani RK, Peyton BM, Magnuson TS (2007). Reduction of cr(VI) under acidic conditions by the facultative fe(lll)-reducing bacterium Acidiphilium cryptum. Environ Sci Technol.

[45] Auch AF, von Jan M, Klenk HP, Goker M (2010). Digital DNA-DNA hybridization for microbial species delineation by means of genome-to-genome sequence comparison. Stand Genomic Sci.

[46] Díaz-Pérez C, Cervantes C, Campos-García J, Julián-Sánchez A, Riveros-Rosas H (2007). Phylogenetic analysis of the chromate ion transporter (CHR) superfamily. FEBS J.

[47] Aguilar-Barajas E, Paluscio E, Cervantes C, Rensing C (2008). Expression of chromate resistance genes from Shewanella sp. strain ANA-3 in Escherichia coli. FEMS Microbiol Lett.

[48] Baaziz H, Gambari C, Boyeldieu A, Ali Chaouche A, Alatou R, Mejean V, Jourlin-Castelli C, Fons M (2017). ChrASO, the chromate efflux pump of Shewanella oneidensis, improves chromate survival and reduction. PLoS One.

[49] Thatoi H, Das S, Mishra J, Rath BP, Das N (2014). Bacterial chromate reductase, a potential enzyme for bioremediation of hexavalent chromium: a review. J Environ Manage.

[50] Belchik SM, Kennedy DW, Dohnalkova AC, Wang Y, Sevinc PC, Wu H, Lin Y, Lu HP, Fredrickson JK, Shi L (2011). Extracellular reduction of hexavalent chromium by cytochromes MtrC and OmcA of Shewanella oneidensis MR-1. Appl Environ Microbiol.

[51] Pushkar B, Sevak P, Parab S, Nilkanth N (2021). Chromium pollution and its bioremediation mechanisms in bacteria: a review. J Environ Manag.

[52] Robins KJ, Hooks DO, Rehm BH, Ackerley DF (2013). Escherichia coli NemA is an efficient chromate reductase that can be biologically immobilized to provide a cell free system for remediation of hexavalent chromium. PLoS One.

[53] Kwak YH, Lee DS, Kim HB (2003). Vibrio harveyi nitroreductase is also a chromate reductase. Appl Environ Microbiol.

[54] Myers CR, Carstens BP, Antholine WE, Myers JM (2000). Chromium(VI) reductase activity is associated with the cytoplasmic membrane of anaerobically grown Shewanella putrefaciens MR-1. J Appl Microbiol.

[55] Li DB, Cheng YY, Wu C, Li WW, Li N, Yang ZC, Tong ZH, Yu HQ (2014). Selenite reduction by Shewanella oneidensis MR-1 is mediated by fumarate reductase in periplasm. Sci Rep.

[56] Beliaev AS, Saffarini DA, McLaughlin JL, Hunnicutt D (2001). MtrC, an outer membrane decahaem c cytochrome required for metal reduction in Shewanella putrefaciens MR-1. Mol Microbiol.

[57] Shi L, Rosso KM, Clarke TA, Richardson DJ, Zachara JM, Fredrickson JK (2012). Molecular underpinnings of Fe(III) Oxide reduction by Shewanella Oneidensis MR-1. Front Microbiol.

[58] Wang C, Chen J, Hu WJ, Liu JY, Zheng HL, Zhao F (2014). Comparative proteomics reveal the impact of OmcA/MtrC deletion on Shewanella oneidensis MR-1 in response to hexavalent chromium exposure. Appl Microbiol Biotechnol.

