# Supplementary material for: Genome analysis of Shewanella putrefaciens 4H revealing the potential mechanisms for the chromium remediation
Source: BMC Genomics. 2024 Feb 2;25:136. doi: 10.1186/s12864-024-10031-9 (PMC10837877; doi:10.1186/s12864-024-10031-9)
Supplement: Supplementary file 1 — Additional file 1: Fig. S1. Morphology and Gram staining of S. putrefaciens 4H colonies(1500x). Fig. S2. Nuclear genome circle diagram. From outside to inside, coding genes (righteous strand), coding genes (negative-sense strand), tRNA (orange) and rRNA (purple), CRISPR, prophage and gene islands, GC ratio, GC-skew, sequencing depth. Fig. S3. Whole genome sequence phylogenetic tree. Fig. S4. Average nucleotide identity (ANI) values of S. putrefaciens 4H with similar model strains. Fig. S5. Gene collinearity analysis of S. putrefaciens 4H and four closely related model strains. Fig. S6. Venn diagram of direct homologous gene between S. putrefaciens 4H and four closely related model strains. Fig. S7. KEGG functional annotation analysis of the core genes. Fig. S8. Functional annotation analysis of COG core genes. Table S1. General features of S. putrefaciens 4H. Table S2. Primers for RT-qPCR experiments. Table S3. DDH values of S. putrefaciens 4H and similar model strains. [file 12864_2024_10031_MOESM1_ESM.docx]

**Fig S1** Morphology and Gram staining of *S. putrefaciens* 4H colonies(1500x)

Fig. S2. Nuclear genome circle diagram. From outside to inside, coding genes (righteous strand), coding genes (negative-sense strand), tRNA (orange) and rRNA (purple), CRISPR, prophage and gene islands, GC ratio, GC-skew, sequencing depth.

**Fig. S3.** Whole genome sequence phylogenetic tree

**Fig. S4.** Average nucleotide identity (ANI) values of S. putrefaciens 4H with similar model strains

**Fig. S5.** Gene collinearity analysis of *S. putrefactions* 4H and four closely related model strains

**Fig. S6.** Venn diagram of direct homologous gene between S. putrefaciens 4H and four closely related model strains


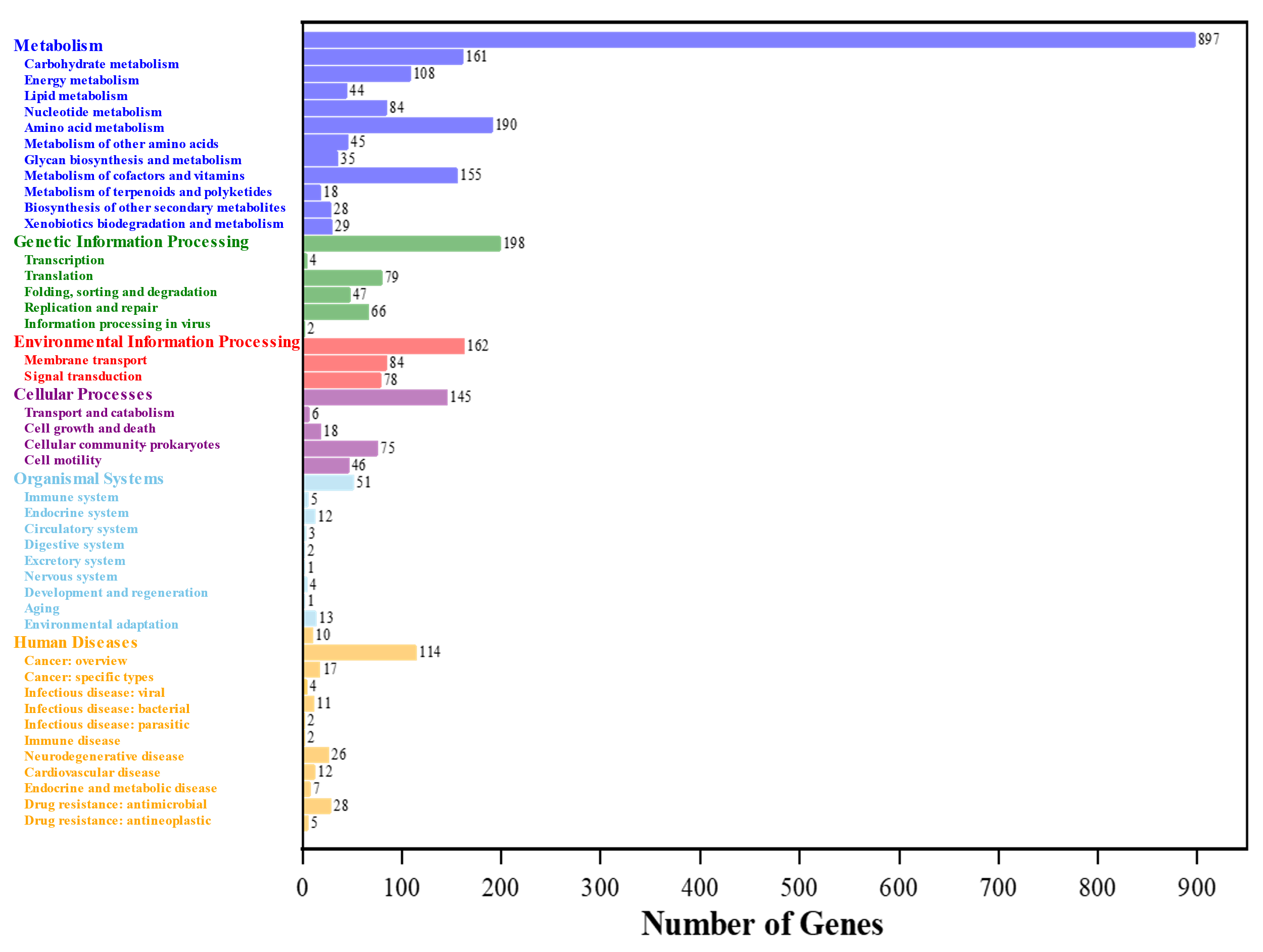


**Fig. S7.** KEGG functional annotation analysis of the core genes


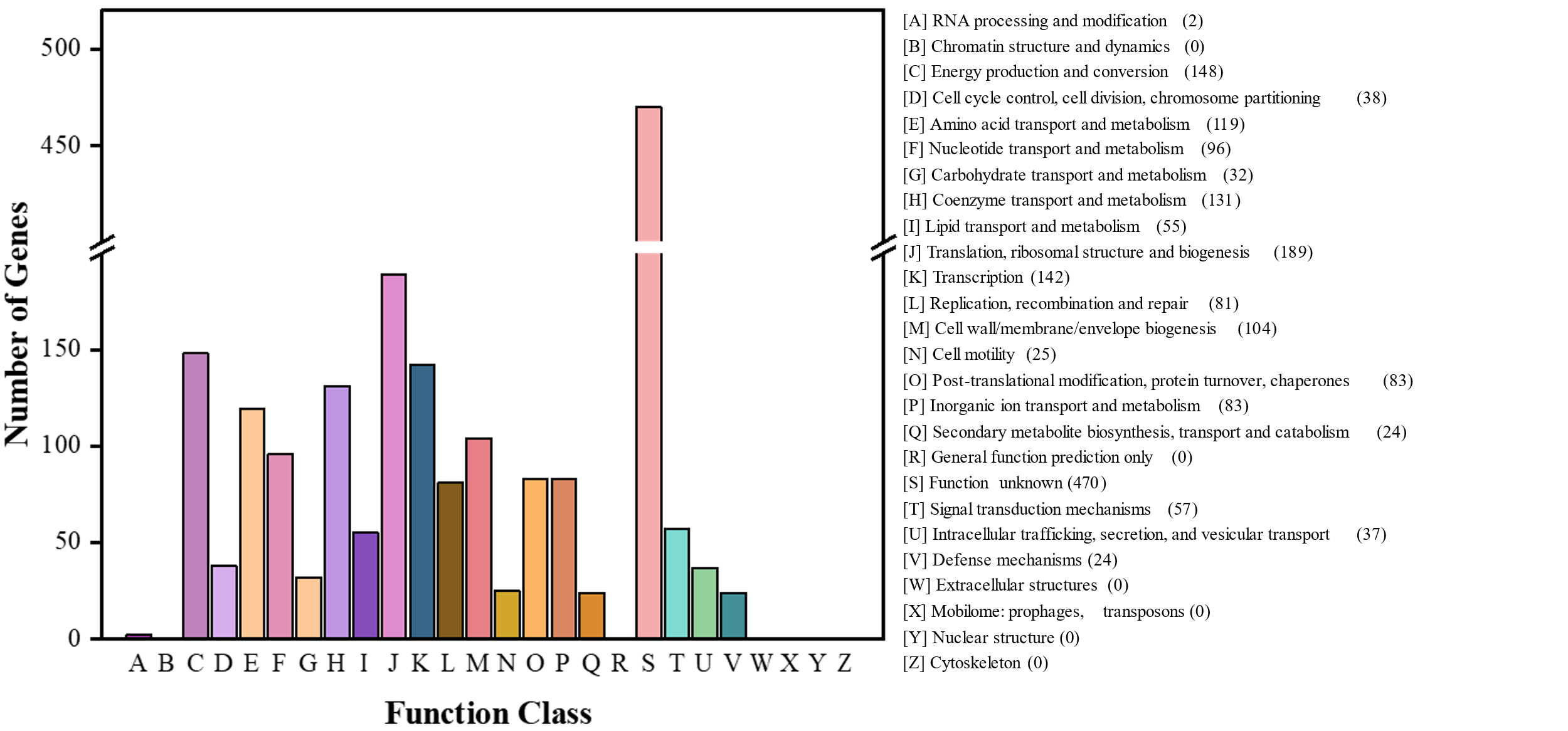


**Fig.S8.** Functional annotation analysis of COG core genes

**Table S1.** General features of *S. putrefaciens* 4H

| Attribute | Value | Attribute | Value |
| --- | --- | --- | --- |
| Genome size (bp) | 4,631,110 | Contigs | 4 |
| GC content (%) | 44.66 | genomic island number | 15 |
| CDS | 4015 | CRISPR repeats | 4 |
| tRNA number | 107 | rRNA number | 25 |

**Table S2.** **Primers for RT-qPCR experiments**

| Primer name | Value |
| --- | --- |
| ChrA-F | GTGGTGGTAGCAGACGCAGTA |
| ChrA-R | TCATTCCCATCCTGAGCACTCA |
| UndA-F | ACGCAGCAGAAGGTGAAGAAGG |
| UndA-R | TCGCTGTGCCATTGTACGGTAA |
| MtrC-F | GTACTGGTACGTTAGTCGATCACA |
| MtrC-R | GCCAGTAAAGACAGGTAGCGT |
| 16S-F | GCCCCCTGGACAAAGACTGAC |
| 16S-R | GACATCGTTTACGGCGTGGACTA |

**Table S3. DDH values of S. putrefaction 4H and similar model strains**

| Strain names | Shewanella putrefaciens 4H |
| --- | --- |
| *Shewanella baltica* OS155 | 26.80% |
| *Shewanella decolorationis* S12 | 22.80% |
| *Shewanella morhuae strain* CW7 | 24.20% |
| *Shewanella oneidensis* MR-1 | 23.20% |
| *Shewanella putrefaciens* CN-32 | 87.30% |
| Shewanella putrefaciens strain SA70 | 23.10% |
| *Shewanella* sp. 11B5 | 22.10% |
| *Shewanella* sp. MR-4 | 22.80% |
| *Shewanella* sp. Pdp11 | 27.00% |
| *Shewanella* sp. POL2 | 23.10% |
| *Shewanella* sp. SNU WT1 | 23.70% |
| *Shewanella* sp. WE21 | 28.90% |
